# Impact of C3 Lowering at Early and Late Stages of Tau Pathology in PS19 Mice

**DOI:** 10.1002/alz70861_108635

**Published:** 2025-12-23

**Authors:** Brijendra Singh, Anouk R Geiser, Ilina Navani, Cynthia A Lemere

**Affiliations:** ^1^ Brigham and Women's Hospital; Harvard Medical School, Boston, MA USA; ^2^ Ann Romney Center for Neurologic Diseases, Brigham and Women's Hospital, Boston, MA USA

## Abstract

**Background:**

Germline depletion of complement C3 has been shown to be protective in a mouse model of tauopathy. Therefore, we asked whether global C3 knockdown at the onset and later in tau pathogenesis would protect against tau aggregation and downstream pathologies later in life.

**Method:**

We generated PS19;C3iKO mice (*PS19^+/−^; C3^fl/fl^; Rosa26‐CreERT2^+/−^*) by crossing P301S tau (PS19) mice with *C3^fl/fl^* and *C3^fl/fl^; Rosa26‐CreERT2* mice. We studied two cohorts: **Cohort 1** received i.p. tamoxifen (TAM; 75 mg/kg) or corn oil (CO) daily for 5 days at **3.6 months of age**. PS19;C3iKO mice received CO (*n = 14*) or TAM (*n = 14*). PS19 control mice received CO (*n = 8)* or TAM (*n = 8*). **Cohort 2** (PS19; C3iKO only) received TAM (*n* =26) or CO (*n* =25) daily for 5 days at **7.5 months**. Behavioral testing was conducted at 9 months of age for cohort 1 and 12 months for cohort 2, after which the mice were euthanized and brain tissue harvested. ELISA and immunofluorescent staining were performed to evaluate the effects of C3 reduction.

**Result:**

Serum C3 levels were significantly reduced ∼85% 30‐days post‐TAM and ∼80% at the end of the study in Cohort 1. Similarly, in cohort 2, serum C3 levels were significantly reduced ∼85% 30‐days post‐TAM and ∼78% at end of the study. Serum C1q levels remained unchanged in Cohort 1 but were significantly reduced in Cohort 2 at the endpoint. No significant changes in locomotion, anxiety, or memory were seen in Cohort 1 mice. Locomotion was increased in the Open Field test, but spatial memory (SNYM) remained unaffected in Cohort 2 mice. No significant differences were observed in brain pathology of pTau (AT8), C1q, GFAP, Iba1, CD68, or Iba1+/CD68+ microglia between TAM‐ and CO‐treated mice. Further biochemical, neuroinflammatory, and synaptic analysis are ongoing to evaluate the therapeutic potential of C3 reduction.

**Conclusion:**

Our data indicates that global C3 lowering in PS19 mice at either the onset or an advanced stage of tauopathy did not alter tau pathology or brain C1q levels. However, late‐stage C3 lowering improved locomotor activity in PS19;C3iKO mice. Funding: NIH/NIA RF1 AG060057 (CAL).

